# Application of Silicon Nanostructure Arrays for 6-inch Mono and Multi-Crystalline Solar Cell

**DOI:** 10.1186/s11671-019-3030-y

**Published:** 2019-06-21

**Authors:** Chen-Chih Hsueh, Subramani Thiyagu, Chien-Ting Liu, Hong-Jhang Syu, Song-Ting Yang, Ching-Fuh Lin

**Affiliations:** 1 Graduate Institute of Photonics and Optoelectronics, National Taiwan University, No. 1, Sector 4, Roosevelt Road, Taipei, 10617 Taiwan; 2 Graduate Institute of Electronics Engineering, National Taiwan University, No. 1, Sector 4, Roosevelt Road, Taipei, 10617 Taiwan; 3 Department of Electrical Engineering, National Taiwan University, No. 1, Sector 4, Roosevelt Road, Taipei, 10617 Taiwan

**Keywords:** Silicon nanowire, Metal-assisted chemical etching, Low reflectance, Six-inch wafer, Carrier lifetime, 81.07.Gf, 78.67.Bf, 78.40.-q, 42.70.Gi

## Abstract

In this study, we fabricate uniform silicon nanowire (SiNW) arrays on 6-inch mono- and multi-crystalline wafers by employing the improved solution-processed metal-assisted chemical etching (MacEtch) method. Furthermore, the improved MacEtch can be applied to various crystalline orientation wafers. The SiNW arrays are 470 nm in length with high density; they demonstrate a good optical trapping effect and reflectance well below 6% over a broad wavelength range from 300 to 1100 nm. The improved MacEtch shows no difference in reflectance for a pyramid/SiNW mono-crystalline wafer with appropriate uniformity; the average delta from the center to other positions is within 22%. The effective lifetime is lower for SiNW arrays because the higher surface state causes higher surface recombination.

Finally, we make the multi-crystalline wafer into an Al-BSF solar cell device with MacEtch SiNW texture, resulting in an averaged power conversion efficiency of 17.83%, which is higher than that of standard acid-textured solar cell devices. Consequently, the improved MacEtch concept is suitable for commercial mass production in the photovoltaic industry.

## Introduction

Recently, silicon nanostructures’ optical properties have attracted tremendous attention due to their excellent light-trapping effect, which results in low reflection and maintains high absorption simultaneously. This effect cannot be found in planar silicon. Silicon nanostructures can be applied on diodes [1, 2], biosensors [3, 4], solar cells [2, 5–13], and etc. In addition, a researcher approximates nanostructures to antireflective layers to explain their light-trapping effect [12]. Therefore, silicon nanostructures can replace traditional costly fabricated antireflective layers.

Much of the scientific literature has investigated the electrochemical characteristics of silicon in fluorine ion solution [13, 14] and utilized the metal-assisted method to fabricate nanostructures in solution to make the processes simple and swift. Therefore, we adopt solution-processed metal-assisted chemical etching to fabricate silicon nanostructures [15]. Unlike molecular beam epitaxy (MBE) [16], laser ablation [17], chemical vapor deposition (CVD) [18], and reactive-ion etching (RIE) [19], which are high-vacuum and high-energy dependent, metal-assisted chemical etching can reduce fabrication costs and can be processed at room temperature.

Moreover, diamond wire sawn (DWS) multi-crystalline wafers have been widely used in solar industries to reduce manufacturing cost, which results in a shining wafer surface, making it hard to maintain the appropriate reflectance through traditional acid texture. Some researchers use an acid texture with extra additives [20]. Also, the RIE texturing method has been studied for aluminum back surface field (Al-BSF) solar cells for reducing reflectance [21].

By utilizing the metal-assisted chemical etching method to fabricate silicon nanostructures, we can control the solution’s oxidant concentration to determine the silicon nanostructures’ etching direction and control the deposited pattern of metal to achieve the nanostructures’ required aspect ratio [14, 15]. The surface orientations and doping levels will also affect the formation of SiNW [22].

Therefore, employing solution-processed metal-assisted chemical etching to fabricate silicon nanostructures is advantageous due to its low cost, simple process, and controllable structure. That is, it is very suitable for commercial practical applications. However, in the literature, solution-processed metal-assisted chemical etching to form silicon nanostructures is only usable on a small area (e.g., ≤ 4 × 4 cm^2^) [9, 22, 23]. Therefore, this research focuses on the uniformity issue on 6-inch wafers. We explore a new approach and investigate the mechanisms for successfully fabricating silicon nanostructures on commercial 6-inch P-type mono-crystalline and p-type multi-crystalline wafers with very high uniformity and low reflection through an improved metal-assisted chemical etching methodology. We also examine nanostructures’ morphologies and optical characteristics to further prove their potential and feasibility for future industrial-oriented commercial applications.

Finally, a 6-inch DWS multi-crystalline p-type nanostructured Si wafers are subjected to synthesized p-n junction aluminum back surface field (Al-BSF) solar cells. Furthermore, we compared the solar cell performance with the acid textured reference wafer.

For solar cell current density-voltage characteristic measurement, the devices were illuminated under 1 sun AM1.5G 100 mW cm^−2^ using solar simulator SUN 2000, Abet Technologies, Inc. and measured using Keithley 2400 source meter. Scanning electron microscopy (SEM) pictures of SiNW array textures were observed using LEO 1530 field emission-SEM. The optical reflectance of SiNW arrays was measured using JASCO V-670 UV-V is spectrophotometer with an integrating sphere. Minority carrier lifetime mapping of SiNW arrays was measured by Semilab μ-PCD WT-2000.

## Experimental Methods

### Mechanism for the Formation of Silicon Nanowire Arrays (SiNW) by MacEtch

**Fig. 1:**
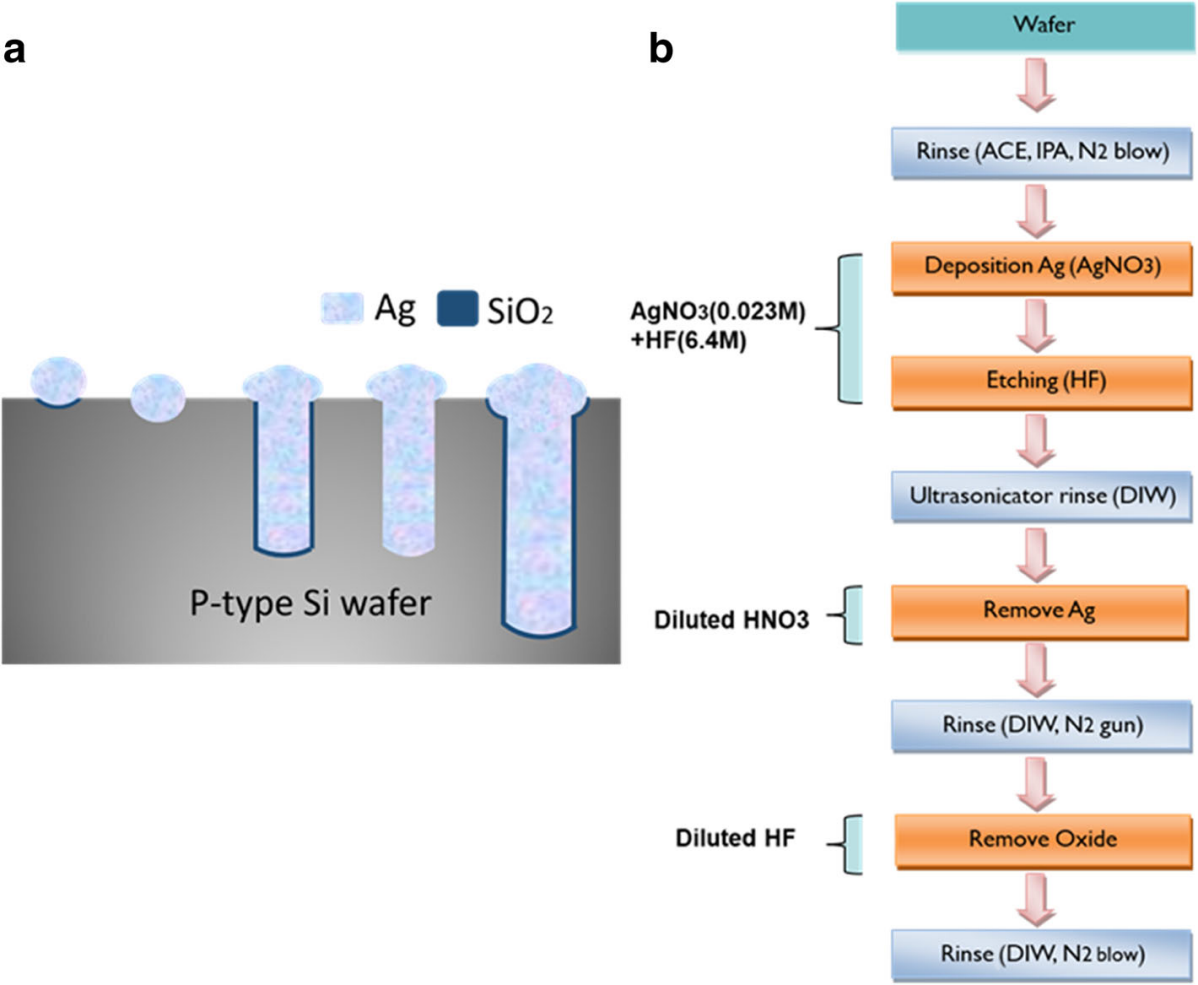
Schematic illustration of the principal of MacEtch method (**a**). Process flow of MacEtch method (**b**)

The method and process flow of MacEtch is shown in Fig. 1a. The etching solution contains silver nitrate (AgNO_3_) and hydrofluoric acid (HF); the Ag^+^ takes the electron from Si and then oxidizes Si into SiO_2_ because the electron negativity of Ag^+^ is larger than that of Si. Moreover, Peng et al. [24] qualitatively compared five metals’ electrochemical potentials and found that the electrochemical potential of Ag^+^ is larger than the valence band of Si. Thus, Ag^+^ will tend to transfer holes to Si and reduce to Ag. In other words, Ag^+^ will take electrons from Si and reduce itself [24]. Therefore, the reduced Ag is deposited on the Si surface, and the surface is oxidized into SiO_2_. Subsequently, the diluted HF is used to remove the oxide. Accordingly, the area with deposited Ag undergoes anisotropic etching, and then, SiNW arrays are formed [22].

### Fabrication of SiNW

In the experiment, the 6-inch with the size of 156 × 156 mm^2^ mm P-type (100) mono-crystalline pyramid-textured wafers and p-type multi-crystalline as-cut wafers were used with a resistivity range of 0.5–3 Ω-cm (thickness 180 + 20/−10 μm). The pyramids’ sizes range from 1 to 10 μm. For the fabrication of silicon nanostructures on 6-inch wafers, the solution process of MacEtch was utilized. The process flow was shown in Fig. 1b. First, the wafers were respectively immersed in acetone, isopropanol alcohol, and de-ionized water (DIW) and cleaned in an ultrasonic bath for 3 min and then dried with a nitrogen blow. Subsequently, the wafers were soaked in an aqueous solution of AgNO_3_, HF, and H_2_O at a ratio of 0.6 g:36 ml:120 ml for 3 min and 19 s at room temperature to etch the SiNW array [13]. The aqueous solution concentration of AgNO_3_ and HF is 23 mM and 6.4 M, respectively, based on the etching condition.

**Fig. 2:**
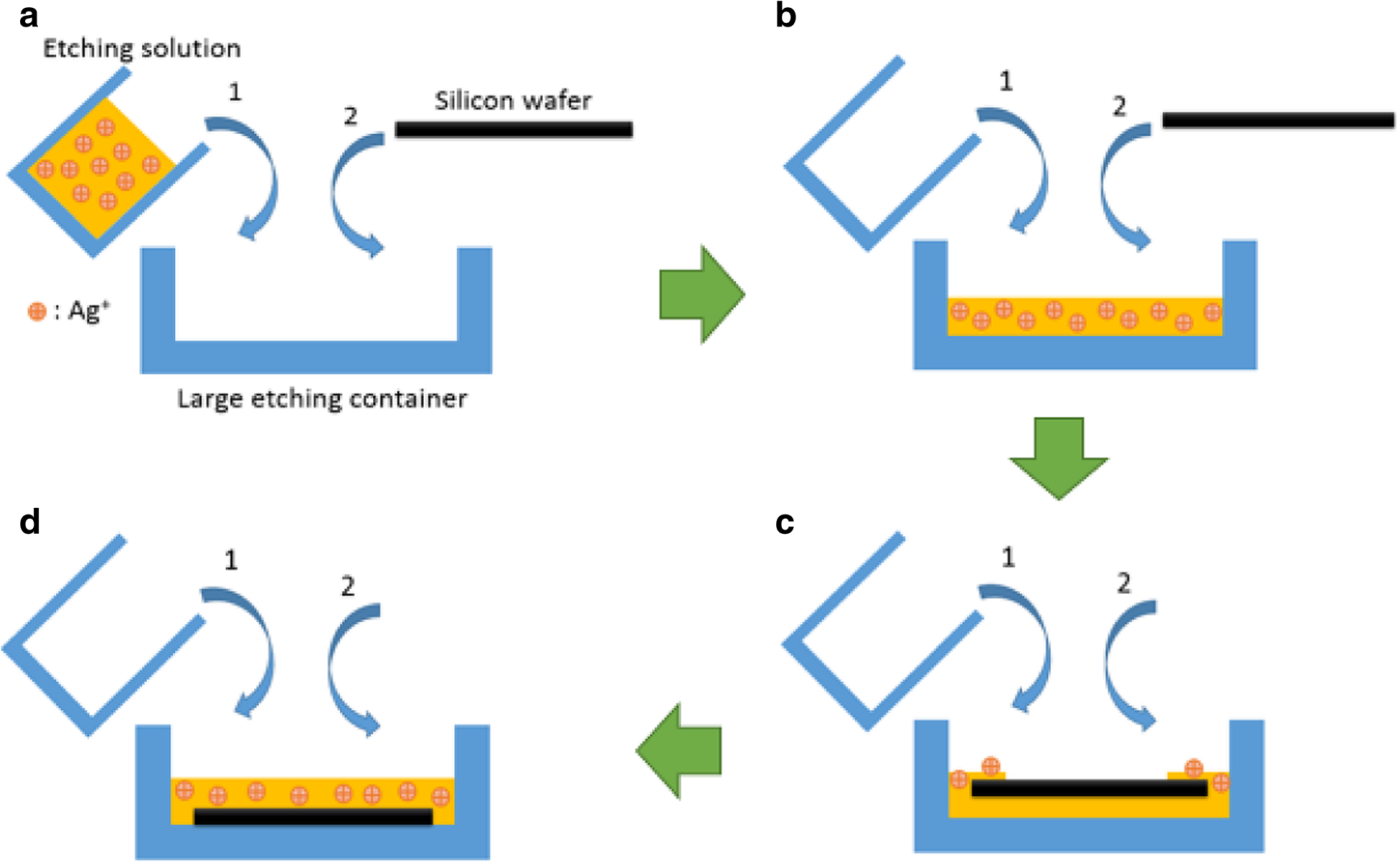
Schematic diagrams of MacEtch steps for method 1 (**a**–**d**)

**Fig. 3:**
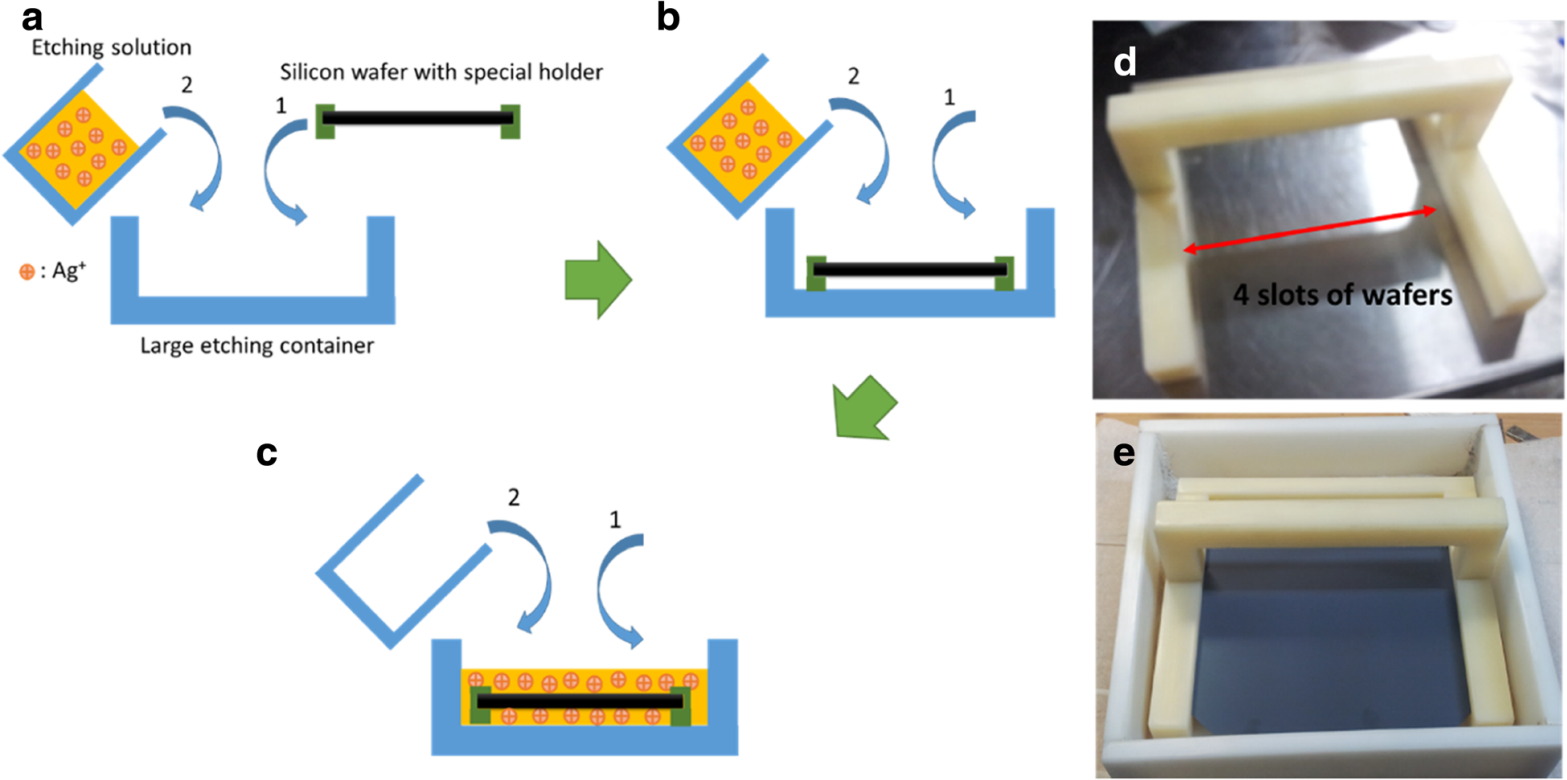
Schematic diagrams of MacEtch steps for method 2 (**a**–**c**). Photos with the special holder (**d**, **e**)

The additional physical influence needs to be considered when the MacEtch method is used to form SiNW on 6-inch silicon wafers, to ensure uniform large-scale SiNW arrays. Subsequently, two fabrication methods are compared. For method 1, the quantitative MacEtch etching solution is first poured into the large etching container, and then, the wafer is placed into the large etching container with the MacEtch solution, which is also a traditional method for small area wafer etching (< 4 × 4 cm^2^) [9, 22, 25] as shown in Fig. 2. For method 2, a modified etching method with a specially designed holder is used for large-scale wafers to achieve large-scale uniform silicon nanostructures and reduce the etching non-uniformity as wafer size increases, and the holder can put 4 pieces of 6-inch wafers, the process flow is shown in Fig. 3. The numbers 1 and 2 noted in the figure represent the sequence of putting etching solution and the silicon wafer, respectively, into large containers. Afterward, the wafers were dipped in a diluted nitric acid (HNO3) solution for 1 min to remove the rest of the silver dendrites. Finally, all the samples were soaked in a diluted HF solution for 1 min to remove surface oxides and then dried with a nitrogen blow.

### Fabrication of 6-Inch Al-BSF Solar Cell

**Fig. 4:**
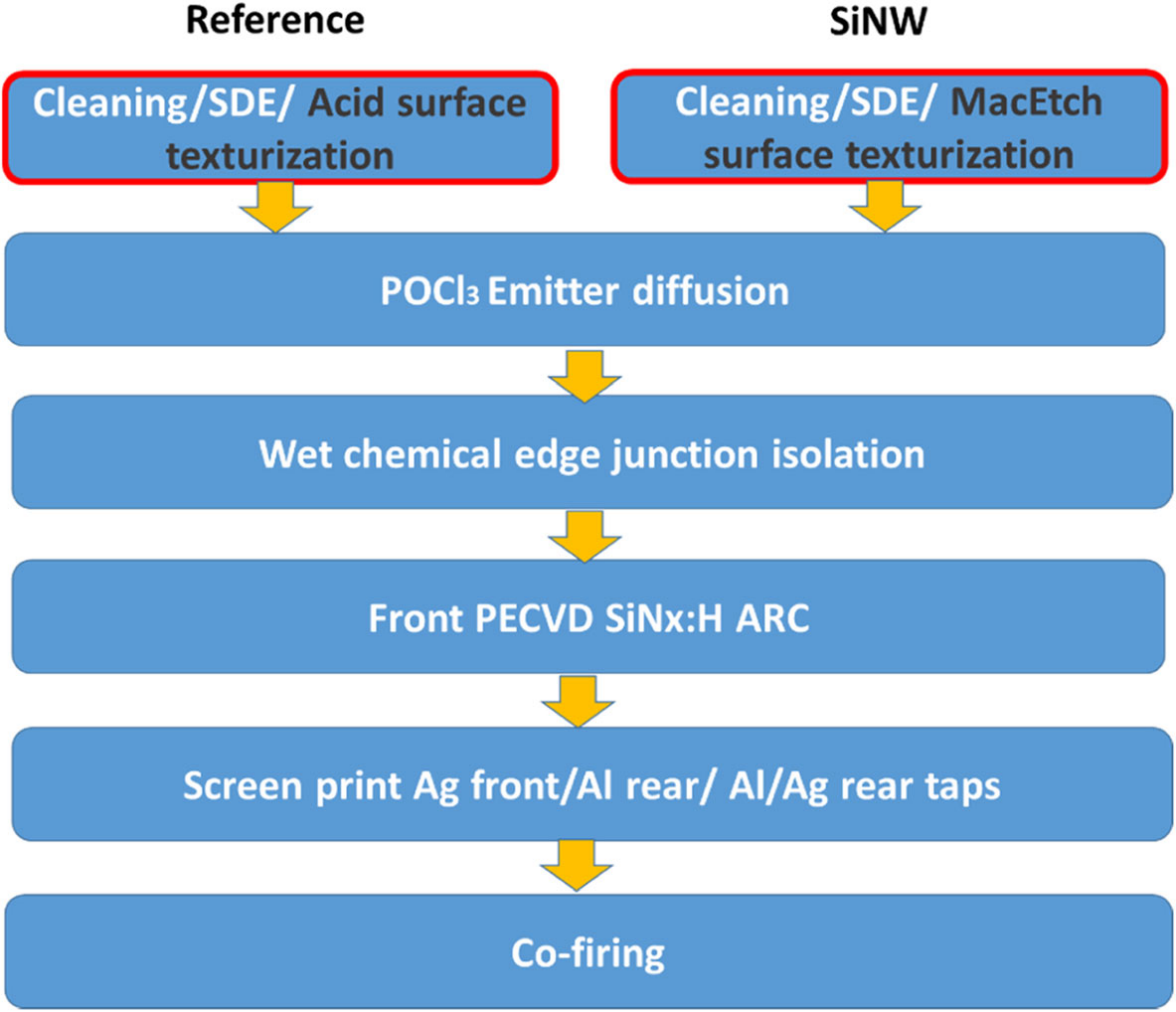
Process flow for the fabrication of conventional industrial screen-printed full area aluminum back surface field (Al-BSF) for both reference and SiNW solar cells

In terms of the Al-BSF solar cell fabrication, we choose multi-crystalline (mc-Si) silicon wafer. The wafer resistivity is 2 Ω-cm, its thickness is 180 μm, and its area is 156 × 156 mm^2^ of size. Figure 4 shows the Al-BSF cell process flow for reference and SiNW [26].

The wafers are cleaned with de-ionized water (DI water), acetone (ACE), piranha solution (H_2_SO_4_:H_2_O_2_), and isopropanol (IPA) for 5–10 min each, saw damage etching (SDE) with 20 wt% KOH solution at 60 °C for 7 min, and a standard HF/nitric acid/acetic acid (HNA) acid solution mixed in the volume ratio 1:3:5 for 5-min texturization as a reference group.

Another test group formed SiNW through MacEtch described in the “Mechanism for the Formation of Silicon Nanowire Arrays (SiNW) by MacEtch” section, a POCl3 diffusion at 850 °C for 30 min to formed N+ emitter layer and the depth was 0.3 mm at the front surface. A sheet resistance of 75 Ω/sq. After the diffusion process, we dipped the silicon wafer into a diluted HF for 5 min to remove phosphorus silicate glass (PSG). A film thickness of ~ 70 nm SiNx:H layer formed from plasma enhanced chemical vapor deposition (PECVD) for anti-reflection coating and passivation, metallization is using the standard Ag-paste and Al-paste screen printing method, front silver and back aluminum electrode, and co-firing successively. In total, there are two groups being proceeded.

## Results and Discussion

### The Method to Achieve Uniform SiNW Arrays on 6-Inch Si Substrates

**Fig. 5:**
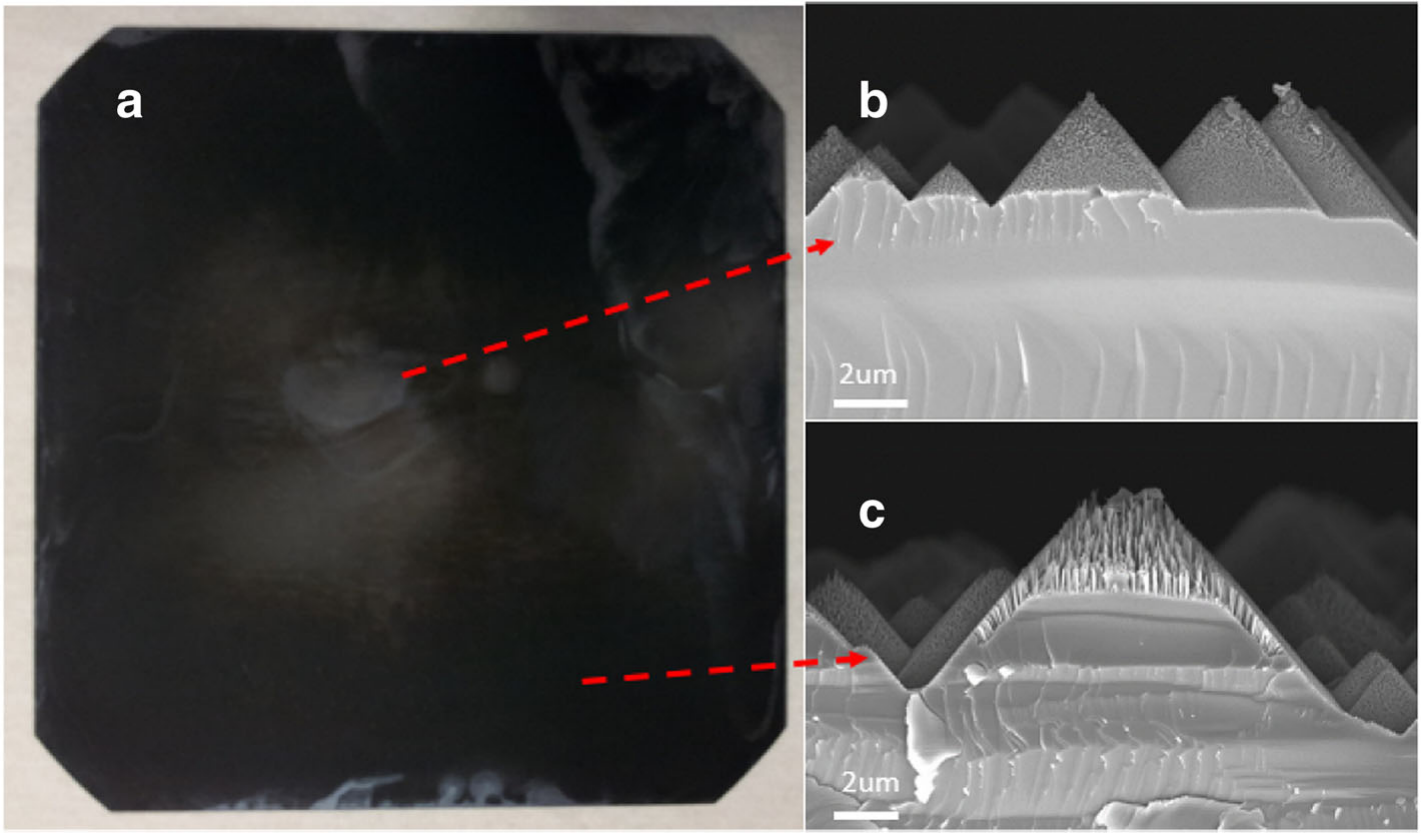
SiNW structure form by method 1. **a** Top view of a 6-inch wafer with SiNW structure. **b** SEM cross-section view of SiNW structure on the center of the wafer. **c** SEM cross-section view of SiNW structure on the corner of the wafer

For method 1, the quantitative silver ion is uniformly distributed first in the large container when the quantitative etching solution is poured into it and place the wafer in the solution Fig. 2b. However, when the large wafer is placed into the etching solution, the etching solution will response a resistance force. This force will cause the etching solution not to immediately uniformly distribute on the wafer’s surface but to slowly diffuse from the wafer’s edges and corners to its center, as shown in Fig. 2c. At this time, the Ag^+^ in the solution starts to react with the wafer’s corner and edge regions, causing the solution’s Ag^+^ concentration to decrease and then causing non-uniform etching of the wafer. Later, despite the remaining concentration of Ag^+^ in the solution, it uniformly distributes on top of the silicon wafer for etching, as shown in Fig. 2d, and the uniform SiNW arrays cannot be obtained. The result is shown in Fig. 5a for the SiNW arrays fabricated from method 1 showing that the SiNW is not uniform. The wafer’s center and corners are investigated by SEM, as shown in Fig. 5b, c. The SEM pictures are all of the same magnification. The wafer already contained a pyramid structure with heights ranging from 1 to 10 μm, so the SiNW structure formed by the MacEtch method on pyramids is investigated. The SiNW arrays around the wafer’s center are shown in Fig. 5b. Only a few SiNW arrays formed. On the other hand, the SiNW arrays near the wafer’s corners are shown in Fig. 5c. The SiNW structure’s depth increases. Hence, inspection and analysis with the naked eye or SEM images reveals that the SiNW arrays formed by method 1 have low uniformity.

In method 1, the Ag+ starts first to react with the corner and the edge of the wafer, which results in the reduction of the concentration of Ag+ in the solution and then causes non-uniform etching of the wafer.

**Fig. 6:**
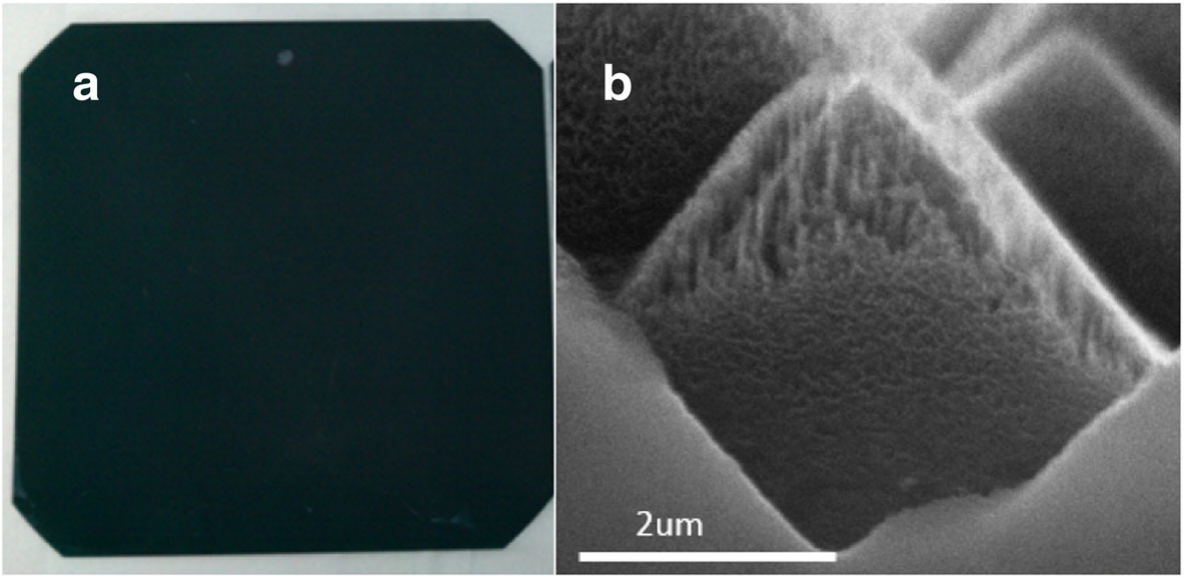
SiNW structure form by method 2. **a** Top view of a 6-inch wafer with SiNW structure. **b** SEM cross-section view of SiNW structure

For method 2, we improve the effect by modifying the MacEtch steps with a holder to increase the surface SiNW uniformity. Subsequently, in this method, the wafer with a holder is put into a large container first, as shown in Fig. 3b, and then, the etching solution is quickly and uniformly poured upon the wafer and container. In this way, the large-scale silicon wafer surface can touch the same concentration of Ag+ at the same time, making the etched SiNW structure uniform. Next, the wafer is soaked in HNO3 to remove the remaining silver dendrites and then immersed in diluted HF to remove surface oxide. The SiNW structures formed by method 2 are shown in Fig. 6a. From the figure, the SiNW structure has good uniformity. The SEM is also used to inspect the surface structure, as shown in Fig. 6b; the length of SiNW is 470 nm^11^ and the density is 3.02 × 1011 cm^−2^.

### Analysis of Etched SiNW Structure and its Surface Uniformity

#### Surface Morphology of 6-Inch Si Wafers

**Fig. 7:**
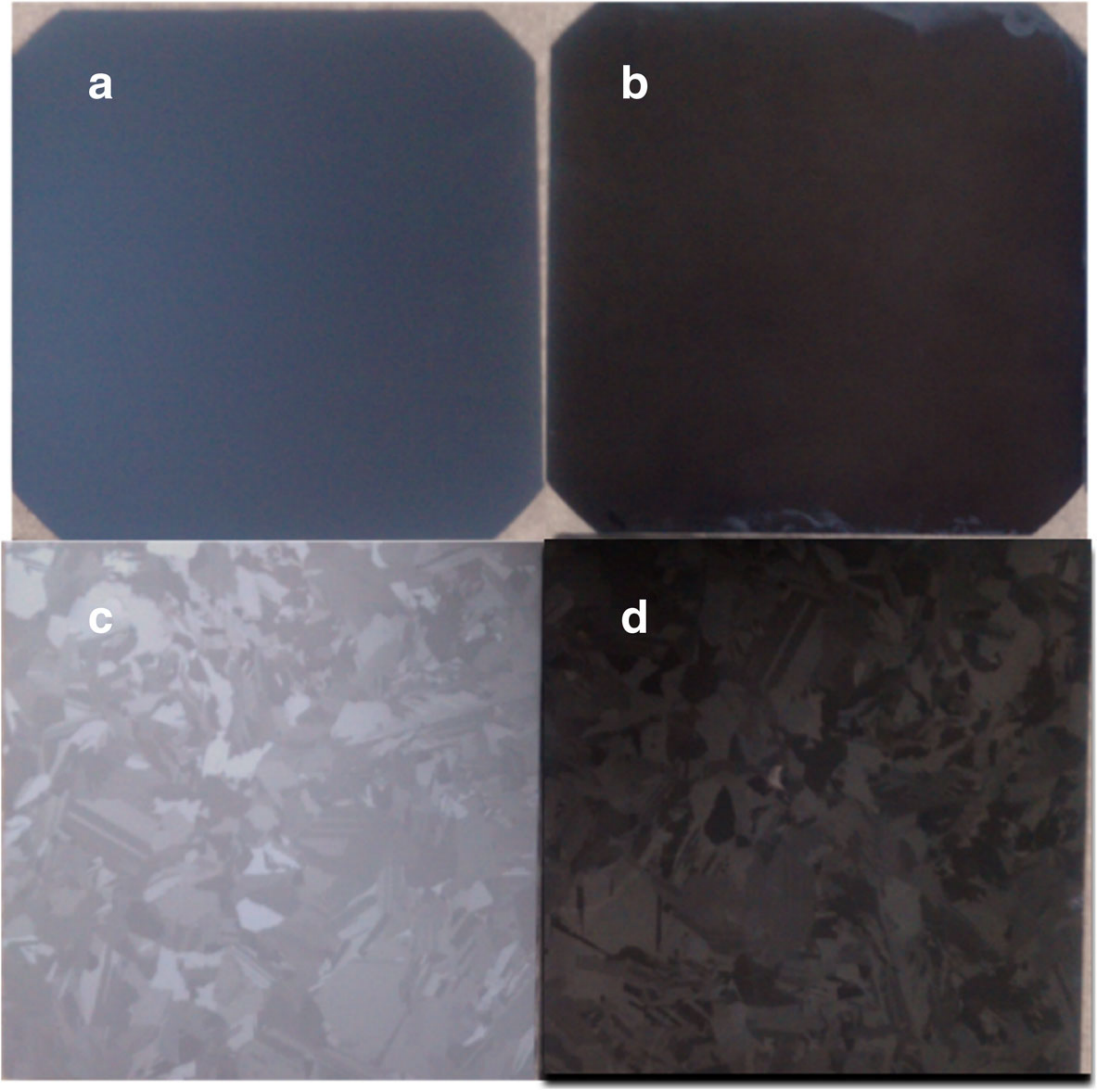
Before and after etched SiNW structure on 6-inch mono-crystalline and multi-crystalline Si wafers. **a**, **b** Before and after etching of mono-crystalline wafers. **c**, **d** Before and after etching of multi-crystalline wafers

We modify MacEtch steps to obtain 6-inch, large-scale, uniform SiNW structures, so as to reduce the effect of time and concentration difference of Ag^+^ contact with the wafer surface. Here, the improved MacEtch method is applied on 6-inch, large-scale, mono-crystalline, and multi-crystalline wafers to fabricate SiNW structures, as shown in Fig. 7. After SiNW arrays are successfully formed on 6-inch wafers, the surface morphology before and after SiNW arrays was formed is investigated. Figure 7 a and c are 6-inch P-type mono-crystalline and multi-crystalline wafers before becoming etched SiNW arrays, respectively. Figure 7 b and d are SiNW arrays formed under the same fabrication condition as described in the experiment. They are formed by using the improved MacEtch method, and the SiNW arrays are etched at the same time. Therefore, the uniform SiNW array structures are successfully fabricated on 6-inch wafers by adopting an improved MacEtch method. In addition, this method demonstrates that it can be applied to different crystal orientation substrates, such as mono-crystal and multi-crystal wafers.

#### SEM Images of SiNW Arrays

**Fig. 8:**
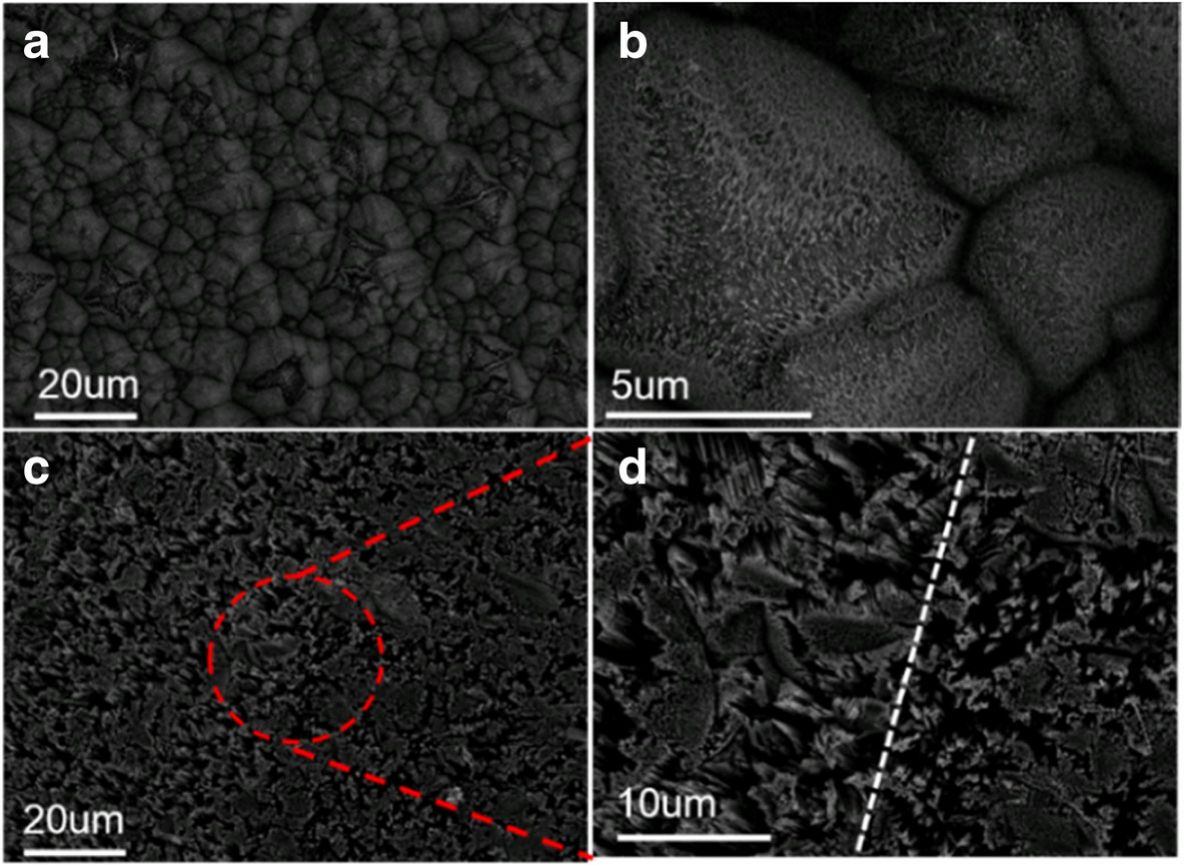
Top view of SEM images. **a**, **b** Pyramid/SiNW array structure on a P-type mono-crystalline wafer. **c**, **d** SiNW array structure on P-type multi-crystalline wafer

The SEM images are used to observe the SiNW morphology on wafer surfaces. Figure 8a is the top view of the pyramid/SiNW array structure formed on a P-type mono-crystalline wafer, and the enlarged pyramid/SiNW array structure is shown in Fig. 8b. It can be clearly observed that the density of SiNW at the top of the pyramid is lower than at the bottom. This is because the top of the pyramid comes in contact with much more etching solution, and then, more Ag metal is deposited on the surface. Therefore, the etched SiNW has lower density.

In contrast, Fig. 8 c and d are SiNW arrays on a P-type multi-crystalline wafer. Indeed, Fig. 8d is the enlarged image of Fig. 8c. For multi-crystalline substrate, the different orientations of SiNW can be clearly observed, and the dimension of a SiNW and its cluster are from 1 to 10 microns. The SiNW is tilted at an angle to the substrate on the left side of Fig. 8d, but SiNW are vertically aligned to the substrate on the right side of Fig. 8d. The back-bond-breaking theory can be used to explain why the MacEtch etching orientation is not vertically aligned to the substrate [15, 20, 22, 25]. A Si atom has two back bonds on the surface of a (100) substrate, but it has three back bonds on the surface of (110) or (111) substrates. In addition, if there are more back bonds, then it is harder to etch or remove. Thus, the Si atom on a (100) substrate is easier to remove, and the etching solution will tend to choose <100> direction for etching, resulting in different orientations of SiNW arrays.

Different orientations of SiNW arrays exhibit different surface colors under naked eye observation, as shown in Fig. 7d. This is because a multi-crystalline Si substrate contains various orientations of crystals, as shown in Fig. 7c, which results in different etched orientations of SiNW and different anti-reflective effects. Moreover, after SiNW arrays are formed on a multi-crystal wafer, the boundaries of different crystal directions can be distinguished by different SiNW orientations, as represented by the dashed line in Fig. 8d.

#### Spectra of Reflectance

**Fig. 9:**
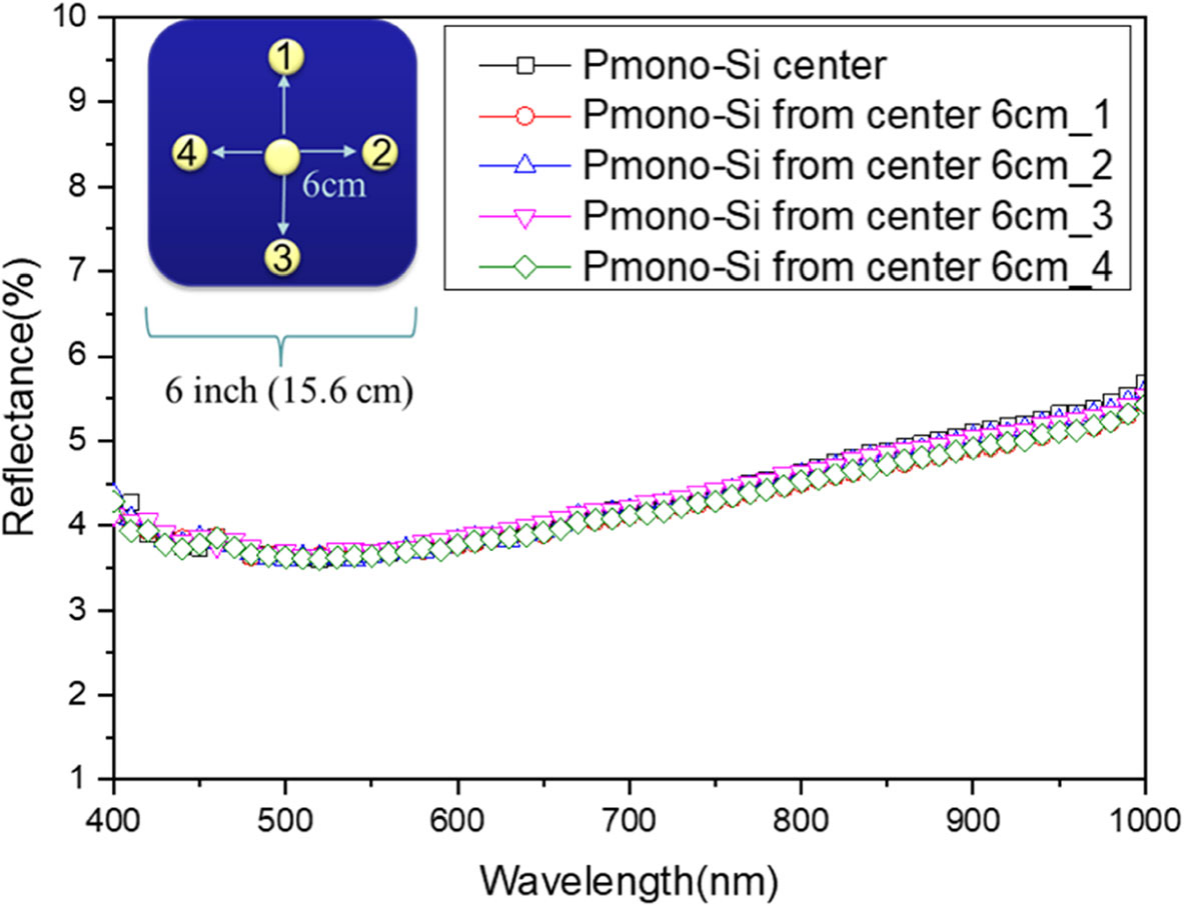
Total reflectance of different spots of 6-inch P-type mono-crystalline pyramid/SiNW array structure wafer. The inset marks the measured area at the center and 6 cm from the center

**Fig. 10:**
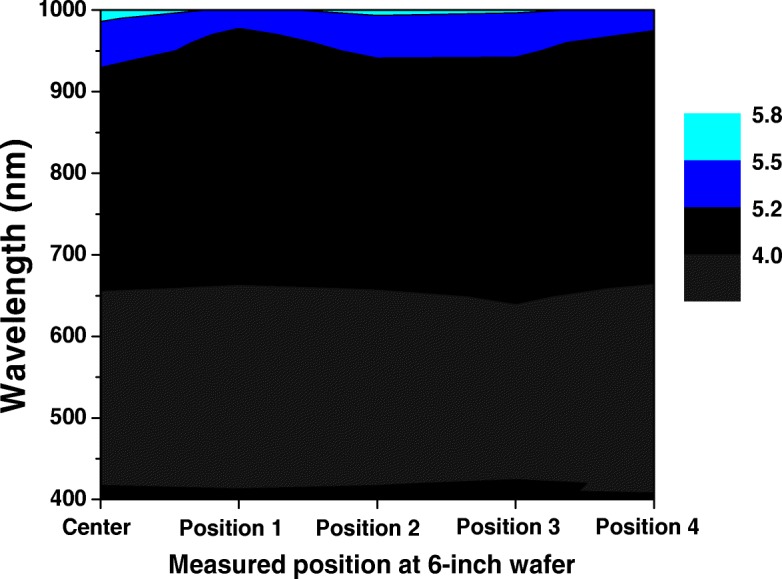
Reflectance mapping of different spots of 6-inch P-type mono-crystalline pyramid/SiNW arrays structure wafer

The optical property of fabricated SiNW arrays is examined here. The reflectance of different spots of a 6-inch P-type mono-crystalline pyramid/SiNW array structure wafer is shown in Fig. 9. The measured spots are at the center and 6 cm from the center of the 6-inch wafer. The optical reflectance of all measured spots is less than 6% for the wavelength range of 400 to 1000 nm, the lowest reflectance is 3% at a wavelength of 500 nm, and the SiNW on the pyramid is with a consistent diameter of 1 micron. This shows that this structure has excellent anti-reflective property. Furthermore, the reflectance mapping for different spots in Fig. 9 is shown in Fig. 10, in which different measured points have almost the same reflectance: the average for the center is 4.358%, position 1 is 4.266%, position 2 is 4.328%, position 3 is 4.263%, and position 4 is 4.265%. The delta is within 22%. This demonstrates that the different spots of 6-inch P-type mono-crystalline pyramids/SiNW arrays have a coherent optical property and, at the same time, also proves that they have very high uniformity by using the improved MacEtch technique to form SiNW arrays.

**Fig. 11:**
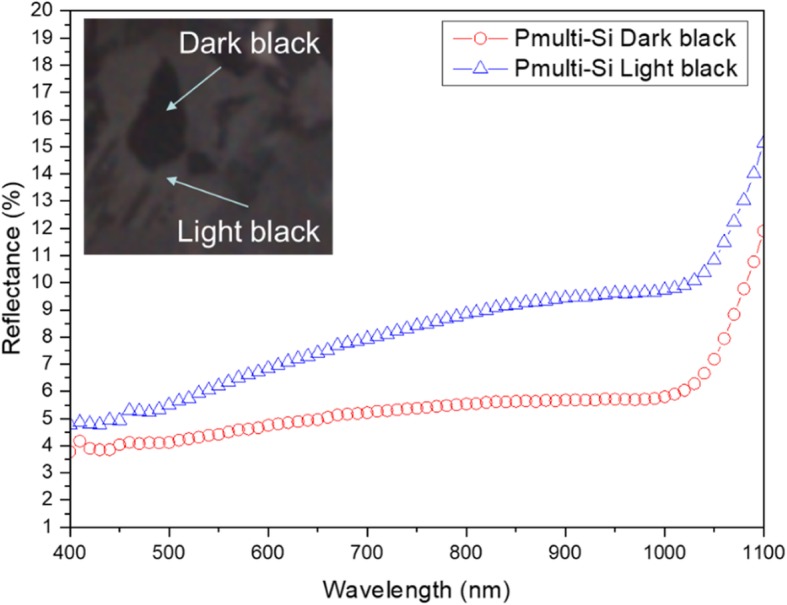
Total reflectance of different spots of 6-inch P-type multi-crystalline as-cut/SiNW array structure wafer

Similarly, the reflectance of different spots of a 6-inch P-type multi-crystalline as-cut/SiNW array structure wafer is measured, as shown in Fig. 11, and it corresponds to the dark black and light black spots in Fig. 7d. The reflectance of the dark black area is lower than that of the light black area. Moreover, the right and left side structures of Fig. 8d can be corresponded to dark black and light black spots, respectively. The reflectance of vertically aligned SiNW arrays is lower than that of SiNW arrays that are slightly tilted to the substrate. This is because vertically aligned SiNW arrays can effectively reflect light several times between SiNW to decrease reflection and increase absorption. Thus, vertically aligned SiNW arrays can keep a good light-trapping property. On the whole, the reflection is lower than 10% from 400 to 1000 nm in wavelength, and the lowest reflection is 4% at 400 nm. Additionally, the reflectance difference of different surface colors is lower than 5%, such as 1% at 400 nm and 5% at 1000 nm for the dark black with SiNW diameter of 1 to 2 μm; for light black with an SiNW cluster from 7 to 10 μm, and average reflectance is approximately 10%. This shows that different orientations of SiNW structure and cluster influence the difference in the light-trapping effect. Furthermore, the maximum reflectance difference for the P-type mono-crystalline pyramid/SiNW array structure in Fig. 9 and the P-type multi-crystalline as-cut/SiNW array structure in Fig. 11 is about 5%. This verifies that the improved MacEtch technique is very suitable for fabricating SiNW array structures on large-scale wafers, regardless if they are mono-crystalline or multi-crystalline silicon.

**Fig. 12:**
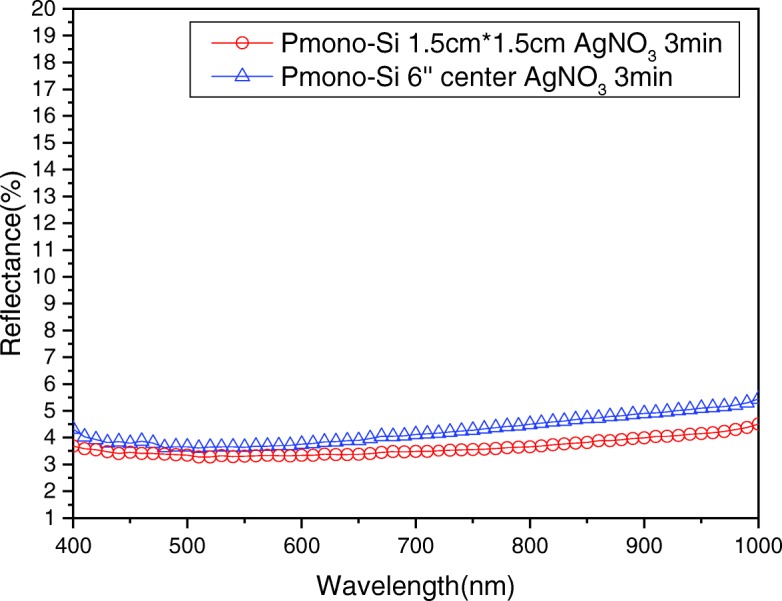
Total reflectance of P-type mono-crystalline pyramid/SiNW array structure formed on 1.5 × 1.5 cm^2^ and 6-inch wafers

In addition, the improved MacEtch steps proposed in this paper are used to create SiNW arrays on different sizes of wafers. Under the same fabrication conditions, the P-type mono-crystalline pyramid/SiNW array structure is formed. The size of wafers is 1.5 cm × 1.5 cm and 6 inches, and then, the reflectance is measured and compared, as shown in Fig. 12, in which the reflection difference is lower than 1%. This shows that we can successfully fabricate almost the same SiNW arrays on large- and small-scale wafers and keep similarly identical optical properties at the same time. Moreover, from Fig. 9, the reflection of different spots of the 6-inch wafers demonstrates that they can maintain high uniformity of SiNW arrays even when the Si wafer size is increased.

#### The Influence of SiNW Structure on Minority Carrier Lifetime

**Fig. 13:**
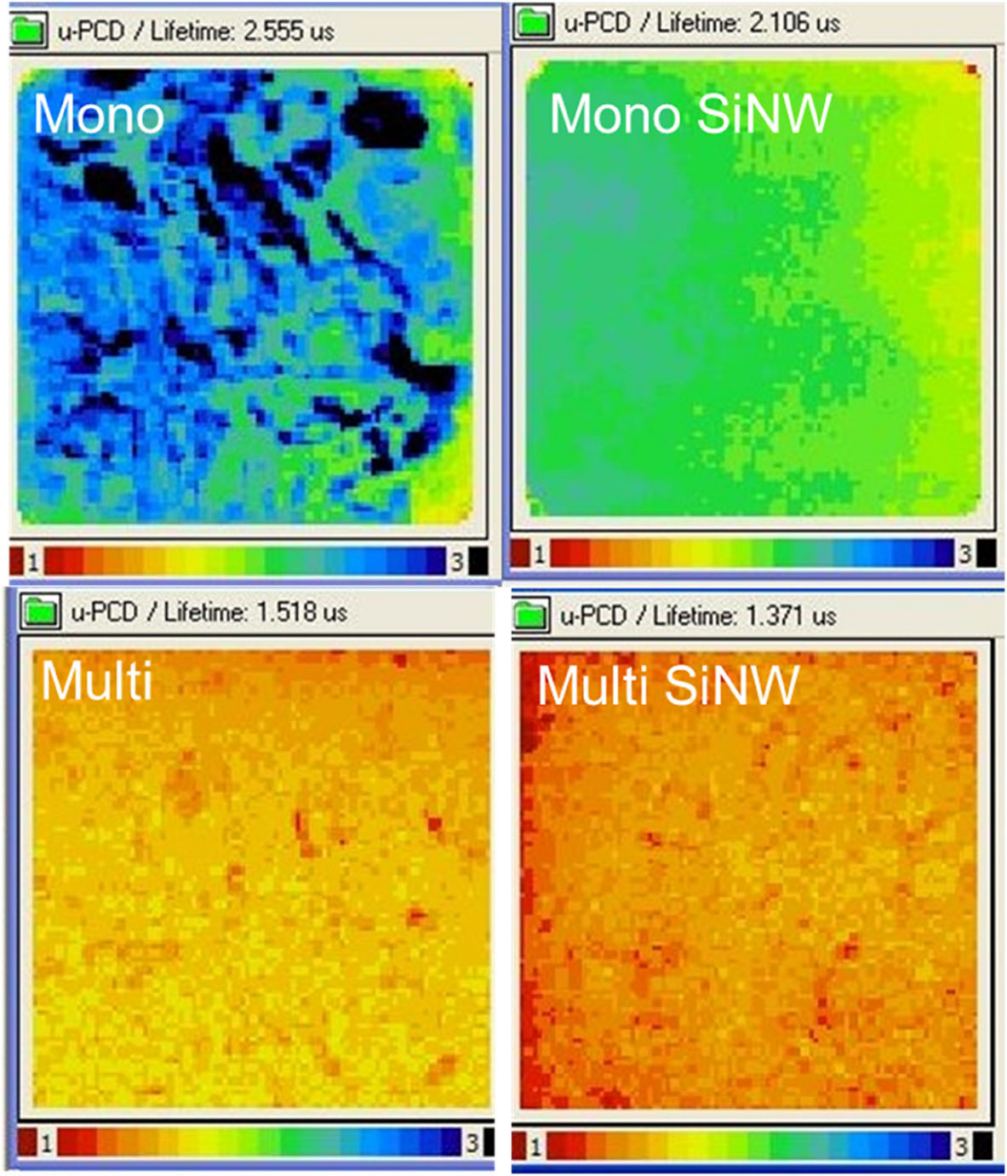
μ-PCD measurement for mapping minority carrier lifetime on 6-inch wafers

Next, we inspected the effect caused by SiNW arrays as surface area changes from a pyramid or as-cut surface to nanostructure arrays. The μ-PCD method is adopted to measure the un-passivated effective minority carrier lifetime of 6-inch P-type mono-crystalline pyramided and P-type multi-crystalline as-cut wafers. The mapping data from before and after fabrication of SiNW arrays are shown in Fig. 13, and the average effective minority carrier lifetime are marked in the figures. The P-mono lifetime slightly decreases from 2.55 to 2.11 μs, and the P-multi lifetime also slightly decreases from 1.51 to 1.37 μs. With either a mono- or multi-P-type, after using the improved MacEtch method to form the SiNW structure, the effective carrier lifetime decreases. This is because the effective surface area is increased due to the etched SiNW on a silicon substrate. Then, the surface recombination probability is increased, which results in a decrease of minority carrier lifetime, as shown in Table 1.

**Table 1:** Average minority carrier lifetime (*τ*_eff_) and simplified surface recombination velocity (*S*_eff_) of different structures

Average	Mono-crystal with a pyramid structure	Mono-crystal with pyramid/SiNW structure	Multi-crystal with bared structure	Multi-crystal with SiNW structure
*τ*_eff_ (μs)	2.555	2.106	1.518	1.371
*S*_eff_ (cm*s^−1^)	3522	4274	5929	6565

From the effective minority carrier lifetime Eq. 1, leads to

1 $$ \frac{1}{\tau_{\mathrm{eff}}}=\frac{1}{\uptau_{\mathrm{bulk}}}+\frac{2{S}_{\mathrm{eff}}}{W} $$

2 $$ {S}_{\mathrm{eff}}\le \frac{W}{2{\uptau}_{\mathrm{eff}}} $$

where *τ*_eff_ is the effective carrier lifetime, *τ*_bulk_ is the bulk carrier lifetime, *S*_eff_ is the effective surface recombination velocity (SRV), and *W* is the wafer thickness.

Because the *τ*_bulk_ is the same for before and after being etched SiNW arrays on N-type mono-crystal or P-type multi-crystal, Eq. (1) can be simplified to Eq. (2) and then the influence of *τ*_bulk_ can be removed. In addition, the wafer substrates are each 180 μm in thickness; hence, from Eq. (2), *S*_eff_ has a negative correlation to *τ*_eff_. Subsequently, for different structures, the average *τ*_eff_ and calculated *S*_eff_ are shown in Table 1 by using the simplified Eq. (2). It can be observed that the *τ*_eff_ has a negative correlation to the simplified *S*_eff_. In conclusion, the etched SiNW arrays can largely increase the surface area of the anti-reflective effect to augment light harvesting. However, SiNW arrays will reduce *τ*_eff_ and increase *S*_eff_ of the wafer, which will reduce the performance of the solar cell. Thus, the effect caused by SiNW arrays should be considered for solar cell applications.

#### Performance of SiNW Multi-crystalline Al-BSF Solar Cell

Regarding solar cell device performance, those cells are measured under AM 1.5G illumination with a power of 100 mW cm^2^ derived from a solar simulator and the cell parameters are summarized in Table 2. Large area SiNW and reference acid-textured solar cell with Al-BSF-based structure were fabricated with an industrially standard cell process, and the averaged test group of SiNW cells achieved 17.83% cell efficiency. Compared to the reference device, the solar cell with SiNWs was about 0.6% gain in efficient, which is a significant gain for an industrial cell. The electrical property of short circuit current density (*J*_sc_), open circuit voltage (*V*_oc_), and fill factor (FF) are also improved. The performance difference is attributed to lower reflectance provided by to SiNWs and leads to a 1.2% gain of *J*_sc_ and 1.35% gain *V*_oc_, which enhances light trapping and absorption in a short wavelength range of 300–400 nm. The FF gain might be attributed to the higher contact area of SiNWs with Al electrodes compared to normal acid-textured surfaces. The efficiency gain can be further improved with a better passivation method for SiNW cells.

**Table 2:** Photovoltaic characteristics of the Al-BSF solar cell device for *R*_ef_ and SiNW structure

Sample type	PCE (%)	*J*_sc_ (mA/cm^2^)	*V*_oc_ (mV)	FF (%)
Reference	17.23 ± 0.09	35.64 ± 0.17	619.78 ± 0.7	77.89 ± 0.06
SiNW	17.83 ± 0.08	36.07 ± 0.16	628.16 ± 0.48	78.46 ± 0.05

## Conclusions

We can successfully use the improved MacEtch steps to fabricate large-scaled SiNW arrays on 6-inch wafers. For the 6-inch P-type mono-crystalline silicon wafer, large-scale uniform and low-reflection pyramid/SiNW array structures can be formed, because the reflection is lower than 6% in wavelengths from 400 to 1000 nm and the lowest reflection is about 3% at a 500 nm wavelength. In addition, experiments have demonstrated that the substrate size has very little influence on the SiNW reflection, which is smaller than 1%. For the 6-inch P-type multi-crystalline silicon wafer, the different surface crystal orientations cause different etching orientations of SiNW arrays and influence the reflection and various surface colors. The reflection is lower than 10% in wavelengths from 400 to 1000 nm, and the lowest reflection is about 4% at a 400 nm wavelength. In addition, the μ-PCD method is adapted to measure the effective minority carrier lifetime of 6-inch P-type mono-crystalline pyramided and P-type multi-crystalline as-cut wafers. We found that the increased surface area of SiNW structures decreases the effective carrier lifetime (*τ*_eff_) of wafers. Here, we use the improved solution-processed MacEtch to form large-scale, uniform SiNW arrays on commercial 6-inch wafers. Regarding cell performance, the device with SiNW arrays has reach averaged of 17.83%, and better *J*_sc_, *V*_oc_, and FF were observed. The improvement is attributed to the SiNW structure’s low reflectance. This process has the advantages of low cost, high compatibility, simplicity, and high throughput. As such, it is very suitable for commercially practical applications in the industry.

## Data Availability

Not applicable

## Abbreviations

FESEM: Field-emission scanning electron microscope
MacEth: Metal-assisted chemical etching
SiNW: Silicon nanowire
μ-PCD FESEM: Microwave photoconductive decay
